# The Complexity of Comorbidity in Patients with Severe Mental Disorders

**DOI:** 10.1192/j.eurpsy.2022.66

**Published:** 2022-09-01

**Authors:** N. Sartorius

**Affiliations:** Association for the Improvement of Mental health Programs (AMH), President, Geneva, Switzerland

**Keywords:** psychiatry, Physical disorders, Management of Comorbidity, comorbidity

## Abstract

Comorbidity of severe mental disorders and physical illness: issues arising Comorbidity of mental and physical illness is a major, perhaps main problem facing medicine in the years before us. In addition to shortening the life expectancy of people with mental illness comorbidity with physical illness comorbidity significantly and negatively affects the quality of life of the people who experience the mental and physical illnesses and their carers and increases the cost of health care. What makes the problem even more and challenging is that medicine is currently in the process of fragmentation into ever more narrow specialties which adds difficulty in the provision of care, Most of the solutions which have been proposed – collaborative care, in-service education of general practitioners and others did not turn out to be effective solutions in dealing with the problems of comorbidity. A significant revision of undergraduate and postgraduate training in medicine is most probably an essential component of the answer to the challenge of this type of comorbidity which will also require a reorganization of health services and their financing.

**Disclosure:**

No significant relationships.

